# The utilization of exosomes in hydrogels: a bibliometric analysis of publications from 2015 to May 2025

**DOI:** 10.3389/fmedt.2025.1657594

**Published:** 2025-09-17

**Authors:** Said El Turk, Abdulrahim A. Sajini, Haider Butt

**Affiliations:** ^1^ Department of Mechanical & Nuclear Engineering, Khalifa University of Science and Technology, Abu Dhabi, United Arab Emirates; ^2^ Department of Biology, Chemistry, and Environmental Sciences, College of Arts and Sciences, American University of Sharjah, Sharjah, United Arab Emirates; ^3^ Advanced Digital & Additive Manufacturing (ADAM) Research Group, Khalifa University of Science and Technology, Abu Dhabi, United Arab Emirates

**Keywords:** exosomes, nanoparticles, hydrogels, polymers, extracellular vesicles

## Abstract

This paper is a bibliometric analysis of the utilization of exosomes in hydrogels for various applications, conducted by assessing the relevant documents in this evolving field of research. Since targeted drug delivery, cell communication, and tissue regeneration are of high importance in the field of biomedicine and medical sciences, incorporating exosomes can offer a valuable addition in such applications due to their high bioactivity and biocompatibility. Applications include ocular drug delivery, boosting optic nerve damage, and disease detection, such as cancer and diabetes. Exosome-based technologies have been of interest since the mid-2000s, with an increased momentum in recent years. In this study, data were exported from the Web of Science and Scopus databases and plotted in order to identify the research trends and publication impact in such an evolving area. The analysis reveals that among several countries, China has the greatest number of publications within the period of 2015 to 9th–11th of May 2025, with a contribution of approximately 76.21% and 61.92% based on the WOS and Scopus databases, respectively. Both publications and citation trends show a significant increase with time, reflecting the increased interest in this field. This study aims to provide an overview of the current impact of research on utilizing exosomes in hydrogel systems.

## 1 Introduction

Exosomes are extracellular vesicles (EVs) secreted by different types of cells and have a size range of 30 nm to 150 nm. They have a significant role in intercellular communication as they transfer biologically active molecules, including lipids, nucleic acids, and proteins, between cells, which influence pathological and physiological processes (1–3). They are naturally created as intraluminal vesicles within multivesicular bodies that are later released into the body fluids, including saliva, urine, and blood (4).

Since exosomes naturally originate from the cells of the human body, they exhibit high biocompatibility, which makes them very applicable in therapeutic applications (5, 6). Their therapeutic applications include them being used as drug delivery vehicles due to their ability to carry and deliver therapeutic molecules to certain targeted cells, which is crucial for treating different diseases such as cardiovascular diseases and neurodegenerative disorders (7). Also, exosomes can carry disease-specific biomarkers, making them beneficial in non-invasive diagnostic applications that can be used to monitor and detect diseases such as cancer (8).

When integrated into hydrogels, the exosomes' stability is improved, and their biological activity is well maintained. Additionally, this allows the release of exosomes into targeted regions in a sustained manner (9, 10). Also, exosome-embedded hydrogels can be used in bone regeneration, as exosomes enhance the hydrogels' regenerative capabilities by regulating inflammatory pathways and promoting osteogenic differentiation (11). Moreover, exosome-loaded hydrogels can be used in improving wound healing as the hydrogels can be used noninvasively on the wounds, providing a conducive region for cell regeneration, which will accelerate the healing rate (12). Also, nanoshells (similar nanostructures like exosomes), such as hyaluronic acid nanoshells, can also be used for drug delivery, where they have been studied in delivering antineoplastic drugs to gliomas and enhancing the performance of contrast agents in nuclear medicine and neuroradiology (13, 14).

In this paper, we analyze the growth of the usage of exosomes in hydrogels by examining the statistics of publications across different fields and countries, providing an overview of the expansion of such research areas worldwide. The statistics discussed in the paper are fully extracted from the Web of Science (WOS) and Scopus databases.

## 2 Materials and methods

This bibliometric analysis paper examined the WOS and Scopus databases in the literature on utilizing exosomes in hydrogels. Both databases allow the advanced search of topics, where a query can be used to target papers of relevance and interest specifically. To maintain consistency between the searches of both databases, the advanced search consisted of identifying the words “exosomes” and “hydrogels” in the titles, abstracts, or keywords of the publications. Where keywords include indexed keywords, keywords plus, and author keywords. To increase the search accuracy, the query was adjusted to detect the aforementioned words in different forms, such as their singulars, plurals, and combinations, including “exosomes in hydrogels”. To ensure that both words are included together in the publications, the “AND” command was used inside the search criteria of abstracts, titles, or keywords without any “OR” command to avoid papers discussing exosomes or hydrogels separately. Both databases enable the extraction of the publication-related information, such as publication dates, authors, and number of citations, which can be used in the analysis. The extracted data was plotted using the Origin software. It is important to point out that the data presented in this review are approximations of very high accuracy. However, complete certainty is not achievable due to the continued increase in publication numbers, citations, and indexing in 2025. Additionally, the counts and proportions for WOS and Scopus were reported separately to avoid overlap, as some publications are indexed in both databases. The VOSviewer software was used to analyse the co-occurrence of keywords extracted from publications in both databases.

## 3 Results and discussion

a. Literature Publication Growth

The data from the databases of Scopus and WOS indicated different numbers of publications, with Scopus giving a higher number of publications. That could be due to the fact that the database of Scopus includes more journals than that of WOS. To minimize the differences as much as possible, an advanced search was used in both databases that searched for the keywords of “exosomes” and “hydrogels” in their various forms in the titles, abstracts, and keywords of the publications. Several publications were thoroughly investigated to ensure that the search resulted in relevant publications, and some were excluded due to irrelevance.

**Figure 1:**
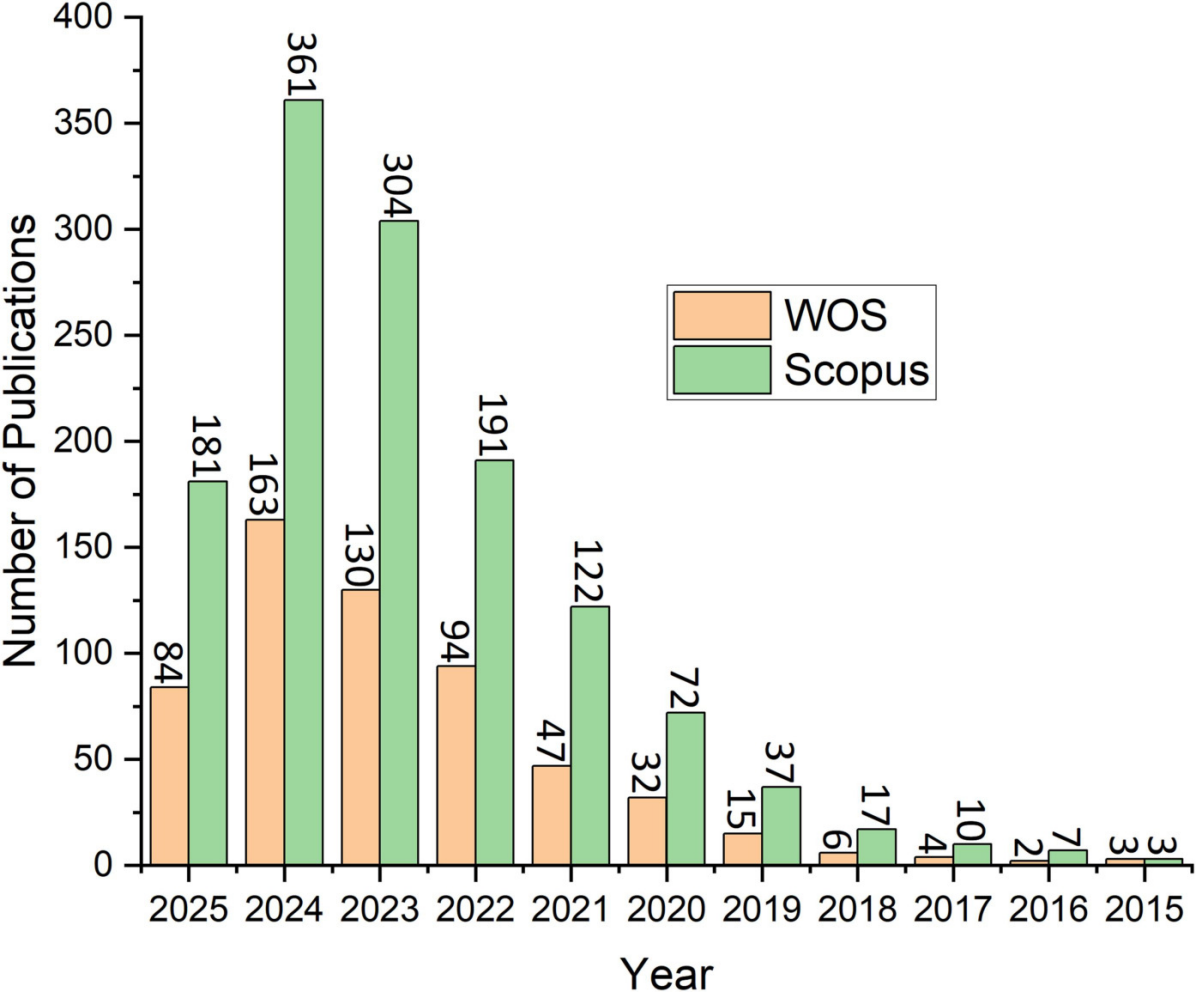
Number of publications per year (up to 9th–11th of May 2025).

As shown in Figure 1, the analysis performed in this study includes publications of the selected types from 2015 to May 9–11, 2025. It is important to point out that initial publications in this field (2–3 publications) in 2013 and 2014 were ignored to maintain consistency between the two databases. In WOS, the highest number of publications recorded was in 2024, with a total of 163 publications accounting for 28.10% of the total selected types publications. Similarly, in Scopus, the highest number of publications recorded was also in 2024, with a total of 361 publications accounting for 27.66% of the total publications. Both databases exhibit a clear, significant increasing trend in publications in recent years, which indicates the increased interest in the subject field.

**Figure 2:**
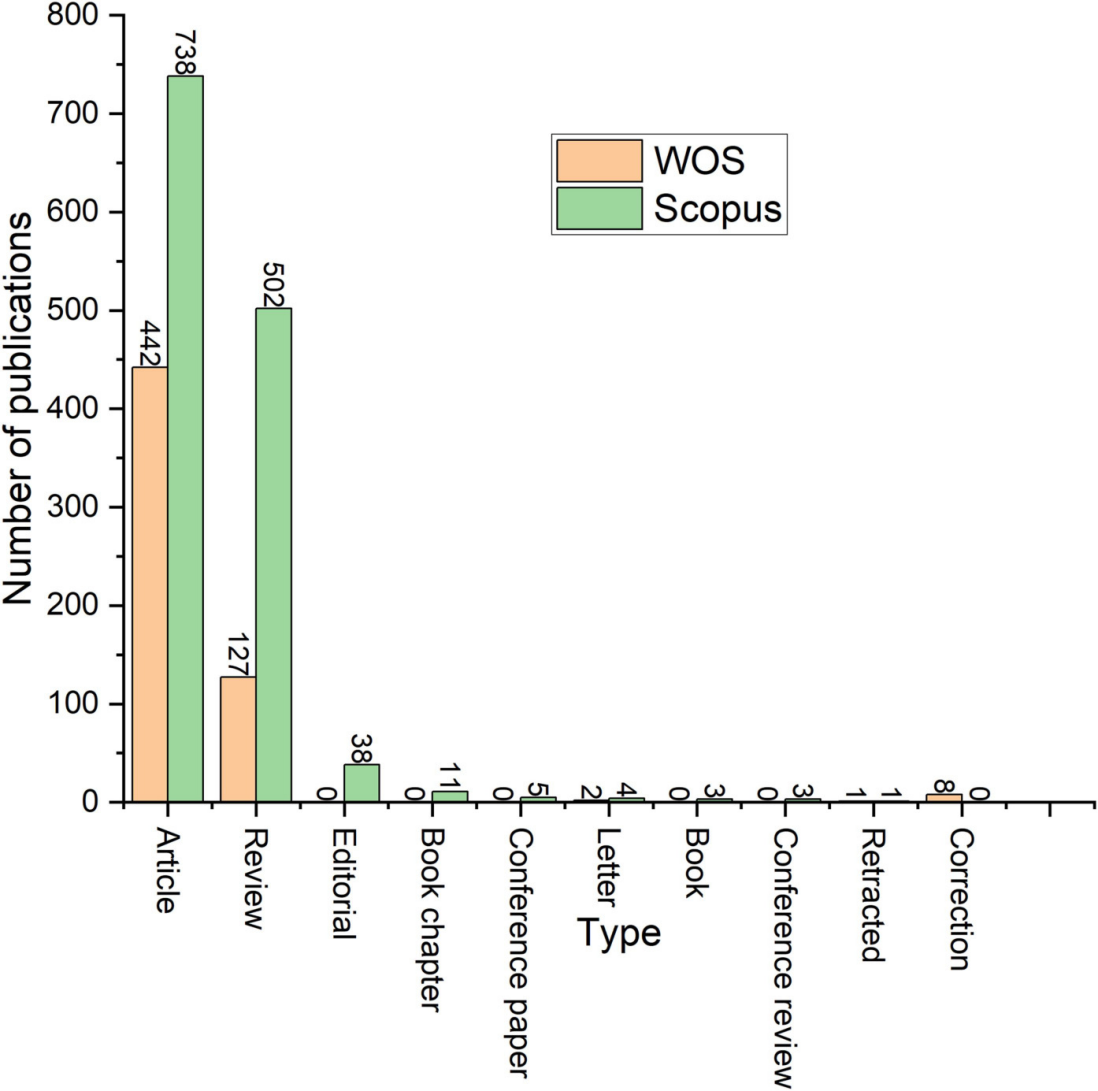
Number of published documents types.

Various types of publications are included in the databases of WOS and Scopus, as shown in Figure 2. This review only includes the publications categorized as articles, reviews, editorials, book chapters, conference papers, letters, conference reviews, retractions, books, and correction, while pre-prints are not included. The types included from WOS and Scopus are only the exact matching terminologies and selected unique terms. A zero value may reflect differences in labeling rather than the absence of publications. The majority of the publications relevant to this field in both databases are scientific articles. The second highest type of publications in both databases are review documents. For instance, the record of articles in WOS is 442 publications, and 738 publications in the Scopus database, accounting for 76.21% and 56.55% of the total publications per database, respectively. Similarly, the record of review papers in the WOS database is 127 publications, and 502 publications in the Scopus database, accounting for 21.90% and 38.47% of the total publications per database, respectively. Hence, the Scopus database comprises of 56.55% research articles and 38.47% review papers. The WOS database comprises of 76.21% research articles and 21.90% review papers.

As seen in Figure 2, Scopus covers document types that are not covered in the WOS database such as conference papers and conference reviews. This could be another reason for the difference in the number of publications between the two databases.

Although publication trends have been showing a significant increase in numbers, the number of clinical trials and patents remains low. An online search outside Scopus and WOS, as they do not index such documents, revealed that very few clinical trials and patents relative to publications exist. Such gaps between research output and translational activity are common in biomedical and biological fields, as it has been reported in areas such as nanomedicine for neuro-oncology (15).

**Figure 3:**
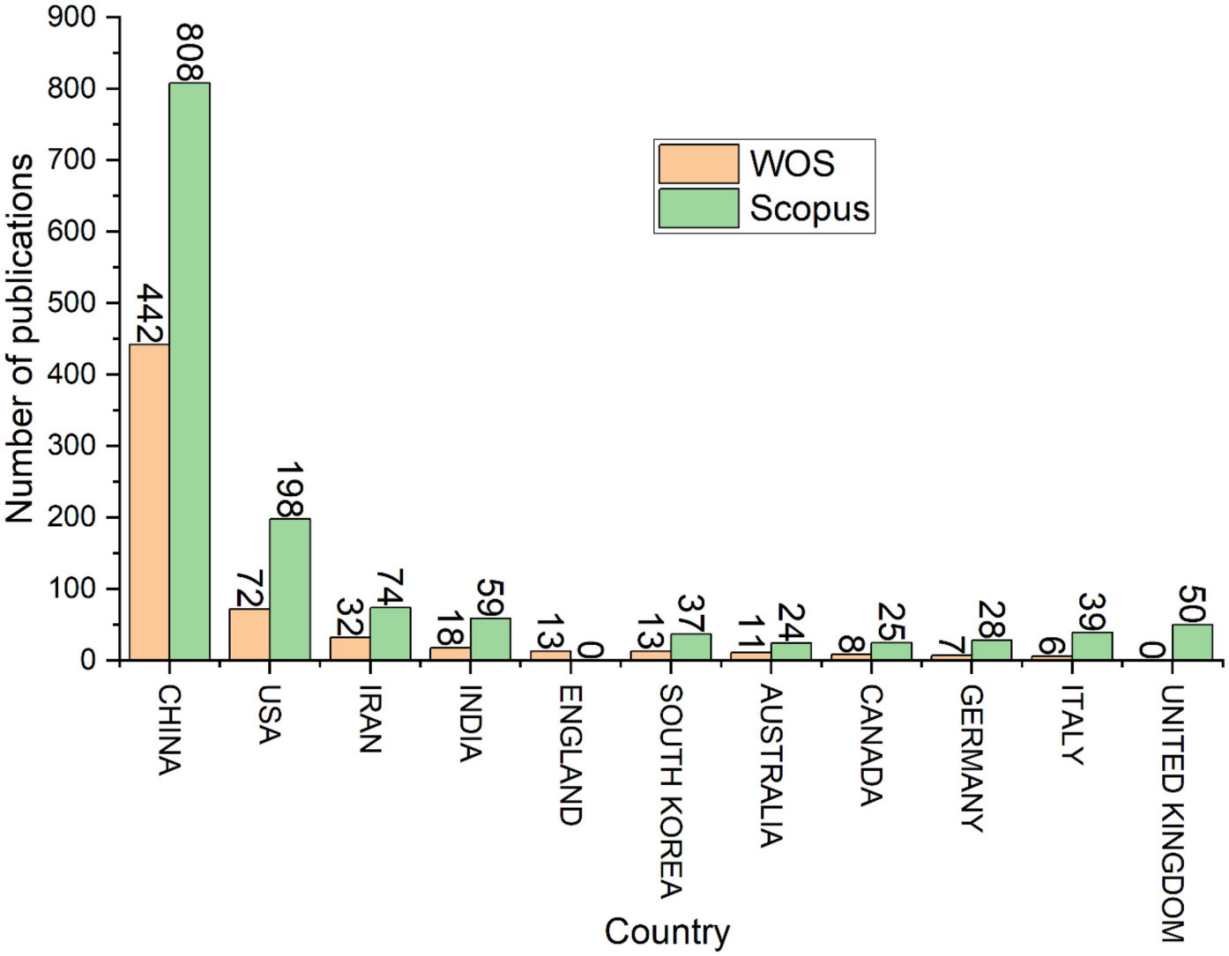
Top 10 countries with the most publications.

Research about exosomes and hydrogels was contributed by several countries (Figure 3), including but not limited to China, Saudi Arabia, the United States of America (USA), England, and Iran. According to both databases, China is the most significant contributor to the research in this field, with 442 publications recorded in WOS and 808 in Scopus, accounting for 76.21% and 61.92% of the total publications, respectively. Both databases rank the top 4 countries as follows: China, USA, Iran, and India. The lowest-ranked country in the top 10 list in the WOS database is Italy, with only 6 publications accounting for 1.03% of the total number of publications. On the other hand, Scopus ranks Australia the lowest of the top 10 countries, with a contribution of 24 publications accounting for 1.84% of the total number of publications.

According to the Scopus database, there are around 159 funding agencies recorded in this field. However, the database of WOS indicates a larger number of funding agencies. The top 10 funding agencies according to the WOS and Scopus databases are listed in Tables 1, 2, respectively. It is important to point out that several agencies are commonly listed in the top 10 agencies recorded in both databases.

**Table 1:** Top 10 funding agencies according to WOS.

No.	Funding agencies	Country/region	Number of publications
1	National Natural Science Foundation of China NSFC	China	290
2	National Key Research Development Program of China	China	49
3	Fundamental Research Funds for The Central Universities	China	30
4	China Postdoctoral Science Foundation	China	28
5	National Institutes of Health NIH USA	USA	26
6	United States Department of Health Human Services	USA	26
7	Guangdong Basic and Applied Basic Research Foundation	China	19
8	National Natural Science Foundation of Guangdong Province	China	19
9	National Key R D Program of China	China	16
10	Beijing Natural Science Foundation	China	13

**Table 2:** Top 10 funding agencies according to Scopus.

No.	Funding agencies	Country/region	Number of publications
1	National Natural Science Foundation of China	China	529
2	Ministry of Science and Technology of the People's Republic of China	China	456
3	National Key Research and Development Program of China	China	114
4	National Institutes of Health	USA	97
5	U.S. Department of Health and Human Services	USA	78
6	China Postdoctoral Science Foundation	China	56
7	Guangdong Provincial Department of Science and Technology	China	50
8	Ministry of Education of the People's Republic of China	China	48
9	Fundamental Research Funds for the Central Universities	China	46
10	European Commission	EU	41

As indicated in Table 1, the funding agency recorded in the WOS database with the highest number of publications is the National Natural Science Foundation of China NSFC, with a total of 290 publications. The highest-ranked agencies from the USA were the National Institutes of Health NIH USA and the United States Department of Health and Human Services. These were ranked at 5th and 6th positions with 26 publications each. The lowest-ranked Chinese agency was the Beijing Natural Science Foundation, with a total of 13 publications. From Table 1, it is clear that the Chinese agencies are the top contributors to research in this field, which reflects their advancement and high interest in the area.

As listed in Table 2, the funding agency recorded in the Scopus database with the highest number of publications is the National Natural Science Foundation of China, with a total of 529 publications. The highest-ranked agencies from the USA were ranked at 4th and 5th positions, with a total number of publications of 97 and 78, respectively. The lowest-ranked Chinese agency is the Fundamental Research Funds for the Central Universities, with a total of 46 publications. Unlike the records from the WOS database, the Scopus database indicates that the European Commission Agency ranked at 10th position in the top 10 agencies, with a total of 41 publications.

b. Paper Publication

Table 3 lists the top-ranked journals in the databases of WOS and Scopus in terms of the number of publications. When comparing the journals extracted from both databases, several journals are ranked high in both databases, but with different numbers of recorded publications. According to the WOS database, the Journal of Nanobiotechnology ranks first with a total of 30 publications. Similarly, the Scopus database ranks the Journal of Nanobiotechnology first with a total of 54 publications. The WOS database ranks the International Journal of Biological Macromolecules and Bioactive Materials 2nd and 3rd^,^ while they are ranked 3rd and 2nd in the Scopus database, respectively. The lowest ranked journal in the WOS database was ACS Applied Materials Interfaces with a total number of publications of 16, while the lowest ranked journal in the Scopus database was the Journal Of Drug Delivery Science and Technology with a total of 29 publications. While, the databases share several top-ranked journals, there are some journals included in one of the databases only, such as Advanced Healthcare Materials, Materials Today Bio, and Pharmaceutics.

**Table 3:** Top journals with the highest number of publications.

WOS		Scopus	
Publication title (source)	Number of publications	Publication title (source)	Number of publications
Journal Of Nanobiotechnology	30	Journal Of Nanobiotechnology	54
International Journal of Biological Macromolecules	26	Bioactive Materials	48
Bioactive Materials	25	International Journal of Biological Macromolecules	48
Materials Today Bio	21	Advanced Healthcare Materials	46
Chemical Engineering Journal	20	Pharmaceutics	35
International Journal of Nanomedicine	17	International Journal of Nanomedicine	32
ACS Applied Materials Interfaces	16	Journal Of Drug Delivery Science and Technology	29

**Figure 4:**
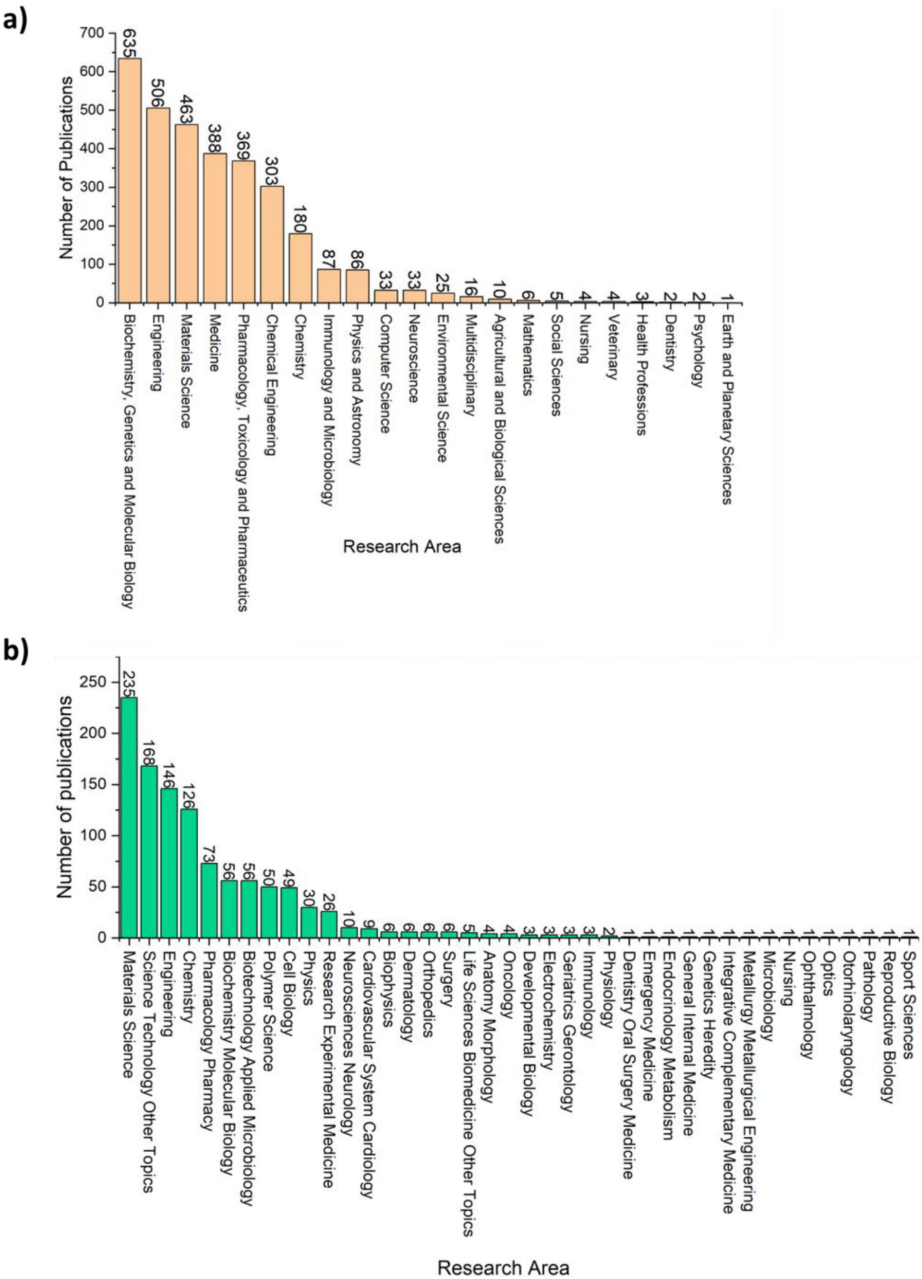
Number of publications based on the research area according to: **(a)** Scopus, **(b)** WOS.

The databases of WOS and Scopus have various categories, with the categories of WOS being more specific while the categories of Scopus are broader (Figures 4a,b). According to the Scopus database, the top three ranked areas are Biochemistry, Genetics and Molecular Biology, Engineering, and Materials Science with 635, 506, and 463 publications, respectively. The lowest-ranked area according to the Scopus database is the Earth and Planetary Sciences field, with only 1 publication.

According to the WOS database, the top three ranked areas are Materials Science, Science Technology Other Topics, and Engineering with 235, 168, and 146 publications, respectively. The lowest-ranked area according to the WOS database was Sport Sciences, with only 1 publication along with several other areas such as Optics, Nursing, Metallurgy, and Pathology.

**Figure 5:**
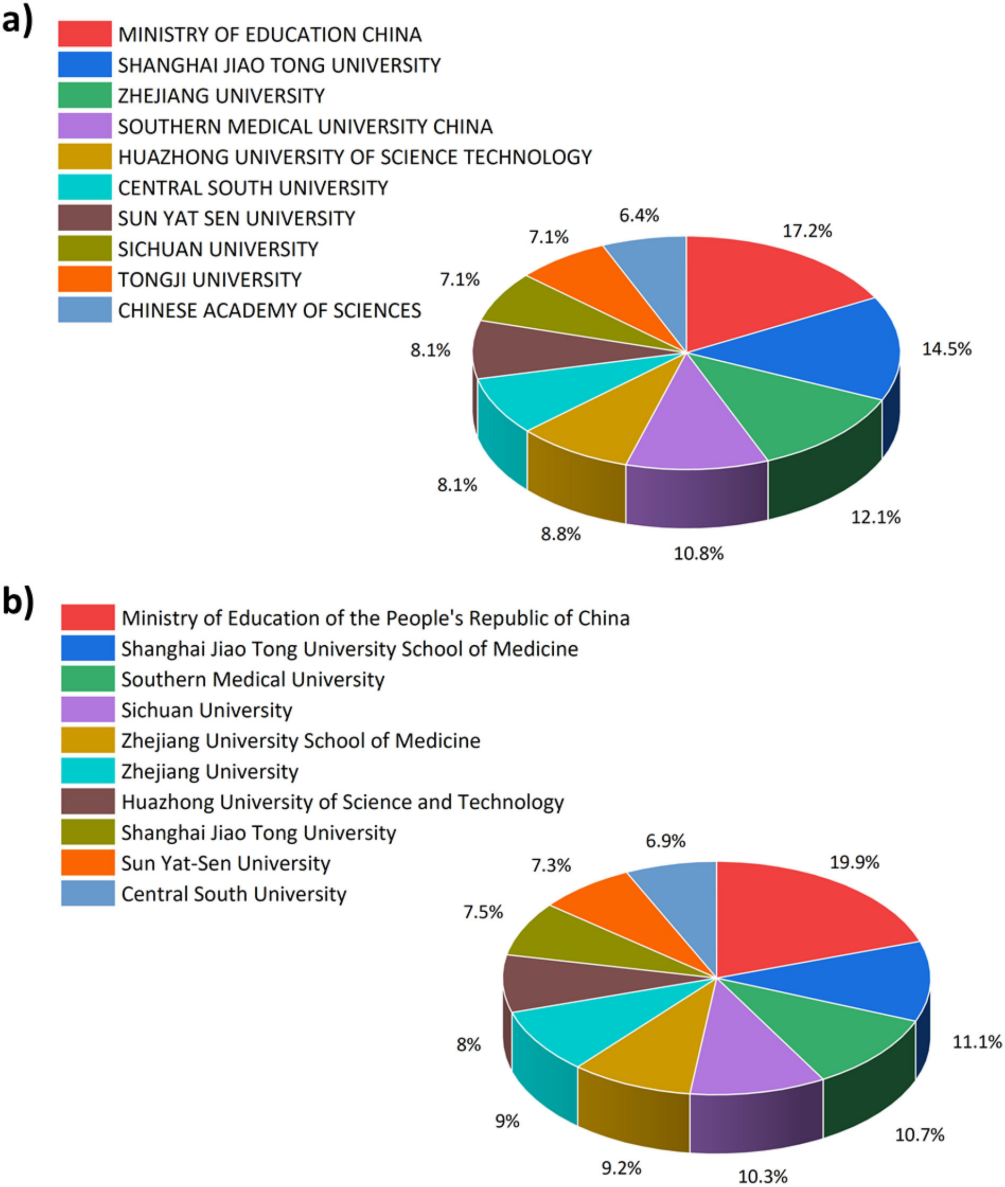
Top 10 organizations listed with the highest number of publications according to: **(a)** WOS, **(b)** Scopus.

Figure 5 shows the top 10 affiliated organizations in terms of the number of publications according to both WOS and Scopus databases (Figures 5a,b). All the top-ranked organizations are Chinese according to both databases. The top three ranked organizations according to WOS are the Ministry of Education China, Shanghai Jiao Tong University, and Zhejiang University, with a list percentage contributions of 17.2%, 14.5%, and 12.1%, respectively. The Scopus database ranks the same 1st and 2nd organizations, but ranks the Southern Medical University as third with a contribution of 10.7% to the list. Most of the organizations are common in both databases, with changes in the order of ranking.

c. Preferred Publications

The WOS and Scopas databases share seven out of the ten most cited publications on the topic of utilizing exosomes in hydrogels. It is important to point out that one publication was omitted from the list of Scopus due to its irrelevance. The omitted paper discusses tumor-associated macrophages immunotherapy, but was included in the search due to the words “exosome” and “hydrogel” being in the indexed keywords. The top-ranked publications presented in Table 4 consist of research articles and review papers, with the majority originating from China, while 1 publication originates from each of the USA, USA/France collaboration, and USA/UK collaboration. The publication with the highest citation count is titled “Engineering Bioactive Self-Healing Antibacterial Exosomes Hydrogel for Promoting Chronic Diabetic Wound Healing and Complete Skin Regeneration” by Wang et al., published in the Theranostics Journal in 2019. This publication has a citation record of 651 in the WOS database and 692 in the Scopus database. The most recent publication in the list is titled “Extracellular vesicle-loaded hydrogels for tissue repair and regeneration” by Yikun Ju et al., published in the Journal of Materials Bio Today in 2023. This publication has a citation record of 244 in the WOS database. The highest cited publication recognized only in the WOS database is titled “Exosomes derived from miR-375-overexpressing human adipose mesenchymal stem cells promote bone regeneration” by Si Chen et al., published in the CELL PROLIFERATION in 2019. This publication has a citation record of 257 in the WOS database. The highest cited publication recognized only in the Scopus database is titled “Genesis and growth of extracellular-vesicle-derived microcalcification in atherosclerotic plaques” by Joshua D. Hutcheson et al., published in the Journal of Nature Materials in 2016. This publication has a citation record of 307 in the Scopus database.

**Table 4:** Summary of the top 10 cited publications per database.

Article title	Article type	Authors	Journal name	Country	Summary	Year	Citations (WOS/Scopus)		Reference
Engineering Bioactive Self-Healing Antibacterial Exosomes Hydrogel for Promoting Chronic Diabetic Wound Healing and Complete Skin Regeneration	Research Article	Wang et al.	Theranostics	China	An injectable hydrogel that was developed to controllably release exosomes to promote angiogenesis, skin generation, and antibacterial activity for chronic diabetic wound healing.	2019	651	692	(16)
Integration of stem cell-derived exosomes with *in situ* hydrogel glue as a promising tissue patch for articular cartilage regeneration	Research Article	Xiaolin Liu et al.	NANOSCALE	China	A photoinduced hydrogel developed for stem cell-derived exosome delivery and preservation at cartilage defect sites. The hydrogel promoted integration with cartilage, and cell, which enhanced the results of cartilage regeneration.	2017	370	402	(17)
Umbilical Cord-Derived Mesenchymal Stem Cell-Derived Exosomes Combined Pluronic F127 Hydrogel Promote Chronic Diabetic Wound Healing and Complete Skin Regeneration	Research Article	Yang et al.	International Journal of Nanomedicine	China	Umbilical cord mesenchymal stem cells (MSCs)-derived exosomes were integrated with a temperature-responsive hydrogel to accelerate chronic diabetic wound healing and promote angiogenesis.	2020	325	354	(18)
Exosomes from nicotine-stimulated macrophages accelerate atherosclerosis through miR-21-3p/PTEN-mediated VSMC migration and proliferation	Research Article	Jumo Zhu et al.	Theranostics	China	Exosomes derived from nicotin-stimulated macrophages were incorporated into chitosan hydrogel and administered to atherosclerotic plaques. The exosomes enhanced vascular smooth muscle cell (VSMC) growth and movement by regulating PTEN, increasing the development of atherosclerosis.	2019	293	316	(19)
Enhanced Therapeutic Effects of Mesenchymal Stem Cell-Derived Exosomes with an Injectable Hydrogel for Hindlimb Ischemia Treatment	Research Article	Kaiyue Zhang et al.	ACS APPLIED MATERIALS & INTERFACES	China	MSC-derived exosomes were incorporated in an injectable chitosan hydrogel to treat hindlimb ischemia. The hydrogel maintained the stability of exosomal content, improved angiogenesis, and accelerated the recovery of ischemic hindlimbs.	2018	292	314	(20)
Transplantation of Human Mesenchymal Stem-Cell-Derived Exosomes Immobilized in an Adhesive Hydrogel for Effective Treatment of Spinal Cord Injury	Research Article	Liming Li et al.	NANO LETTERS	China	Human MSC-derived exosomes were incorporated in an adhesive hydrogel and implanted at spinal cord injury sites. The results indicated efficient exosome release and preservation, reduced inflammation, and hence, significant nerve recovery.	2020	289	300	(21)
GMSC-Derived Exosomes Combined with a Chitosan/Silk Hydrogel Sponge Accelerates Wound Healing in a Diabetic Rat Skin Defect Model	Research Article	Quan Shi et al.	FRONTIERS IN PHYSIOLOGY	China	Gingiva-derived mesenchymal stem cell (GMSC)-derived exosomes were integrated into a hydrogel sponge applied to diabetic wounds. the results indicated that the system promoted re-epithelialization, angiogenesis, neuronal regeneration, and collagen deposition, introducing a non-invasive exosome administration approach.	2017	260	313	(22)
Genesis and growth of extracellular-vesicle-derived microcalcification in atherosclerotic plaques	Research Article	Joshua D. Hutcheson et al.	Nature Materials	USA/UK	A collagen hydrogel model was used to simulate fibrous caps, where the study monitored the clustering of calcifying EVs, causing micro- and macro-calcifications. The results revealed a mechanistic understanding of plaque destabilization.	2016	307		(23)
Cardiac recovery via extended cell-free delivery of extracellular vesicles secreted by cardiomyocytes derived from induced pluripotent stem cells	Research Article	Bohao Liu et al.	Nature Biomedical Engineering	USA	A hydrogel patch was fabricated to release cardiomyocyte-derived EVs from induced pluripotent stem cells (iPSCs) to damaged hearts. The slow release promoted ejection-fraction recovery, decreased the size of infarctions, and reduced arrhythmias, indicating a therapeutic potential for treating heart injury.	2018	284		(24)
Biomaterials Functionalized with MSC Secreted Extracellular Vesicles and Soluble Factors for Tissue Regeneration	Review	Brennan et al.	Advanced Functional Materials	USA/France	MSCs-derived Evs and soluble factors were integrated into biomaterials to enhance cell survival, angiogenesis, and decrease fibrosis and inflammation for tissue regeneration.	2020	278		(25)
Exosomes derived from miR-375-overexpressing human adipose mesenchymal stem cells promote bone regeneration	Research Article	Si Chen et al.	CELL PROLIFERATION	China	Exosomes enriched with miR-375 were encapsulated in a hydrogel and applied to a calvarial defect model. The exosomes promoted osteogenic differentiation of hBMSCs by inhibiting IGFBP3. The patch demonstrated slow release of exosomes and enhanced bone regeneration.	2019	257		(26)
Exosomes-Loaded Electroconductive Hydrogel Synergistically Promotes Tissue Repair after Spinal Cord Injury via Immunoregulation and Enhancement of Myelinated Axon Growth	Research Article	Fan et al.	Advanced Science	China	BMSCs-derived exosomes were incorporated into electrically conductive hydrogels to regulate inflammation, facilitate spinal cord injury repair, and promote axon growth.	2022	255		(27)
Extracellular vesicle-loaded hydrogels for tissue repair and regeneration	Review	Yikun Ju et al.	Materials Bio Today	China	A self-healing injectable hydrogel was used to encapsulate and controllably deliver EVs, enabling long-term retention at target tissue sites. Such system can promote wound healing by synergizing EVs, providing an efficient approach for wound healing applications.	2023	244		(28)

Yellow = recognized only in the WOS database, Green = recognized only in the Scopus database, Blue = recognized by both databases.

The top cited publications per database (Table 4) can be categorized using their intended application. Cardiovascular and vascular pathology/repair, as well as skin and soft tissue wound healing categories, contribute the most to the list with a contribution of around 30.8% and 23.1%, respectively. While the musculoskeletal regeneration (bone and cartilage), spinal cord injury treatment, and tissue regeneration/reviews categories contribute equally to the list, with a percentage of 15.4% each.

The top 10 most recent publications in both databases are listed in Table 5. All the publications listed were published in 2025. Notably, around 30% of the publications in the list are published in the Journal of Nanobiotechnology. Also, the Chinese Journal of Tissue Engineering Research and the Bioactive Materials journal each have publications accounting for 10% of the listed publications. The publications from both databases include articles and review papers that investigate different applications, including cardiac repair, burned skin repair, diabetic wound treatment, bone repair, and the treatment of infectious tissue injuries. The top 10 most recent publications per database (Table 5) can be categorized using their intended application. Musculoskeletal regeneration (bone, cartilage, and tendon) as well as the reviews categories, contribute the most to the list with a contribution of around 21.1% and 15.8%, respectively. The spinal cord injury treatment category has a contribution of 10.5%. While the cardiovascular repair and organ repair (kidney) categories contribute equally to the list, with a percentage of 5.3% each. As seen in Table 5, several publications include the utilization of EVs instead of exosomes; this is mainly due to their inclusion of terms such as exosomes and hydrogels in their keywords.

**Table 5:** Top 10 most recent published papers based on Scopus and WOS database.

WOS			Scopus		
Publication title (source)	Year	Reference	Publication title (source)	Year	Reference
Unpacking Exosomes: A Therapeutic Frontier for Cardiac Repair (*Current Cardiology Reports*)	2025	(29)	Deer antler stem cell exosome composite hydrogel promotes the repair of burned skin (*Chinese Journal of Tissue Engineering Research*)	2025	(30)
A dual-action strategy: Wound microenvironment responsive hydrogel and exosome-mediated glucose regulation enhance inside-out diabetic wound repair (*Journal of Controlled Release*)	2025	(31)	Application of exosome-loaded hydrogel in nerve injury regeneration and wound healing (*Chinese Journal of Tissue Engineering Research*)	2025	(32)
Breathable core-shell microneedle patches for diabetic wound treatment (*Materials Futures*)	2025	(33)	Dedifferentiated fat cells-derived exosomes (DFATs-Exos) loaded in GelMA accelerated diabetic wound healing through Wnt/*β*-catenin pathway (*Stem Cell Research and Therapy*)	2025	(34)
Multifunctional magneto-electric and exosome-loaded hydrogel enhances neuronal differentiation and immunoregulation through remote non-invasive electrical stimulation for neurological recovery after spinal cord injury (*Bioactive Materials*)	2025	(35)	Decellularized tissue matrices hydrogels functionalized with extracellular vesicles promote macrophage reprogramming and neural stem cell differentiation for spinal cord injury repair (*Journal of Nanobiotechnology*)	2025	(36)
Methacryloylated chitosan hydrogel-mediated polyphenol-Ga/ hUCMSC-Exo release platform: Possessing antibacterial, anti-inflammatory, and osteogenic capabilities (*International Journal of Biological Macromolecules*)	2025	(37)	RGD hydrogel-loaded ADSC extracellular vesicles mitigate uranium-induced renal injury via TLR4/NF-*κ*B pathway inhibition (*Journal of Nanobiotechnology*)	2025	(38)
GelMA/HA-NB hydrogel encapsulating adipose-derived chondrogenic exosomes enhances enthesis regeneration in chronic rotator cuff tears (*International Journal of Biological Macromolecules*)	2025	(39)	Engineered extracellular vesicles with sequential cell recruitment and osteogenic functions to effectively promote senescent bone repair (*Journal of Nanobiotechnology*)	2025	(40)
Restoring tendon microenvironment in tendinopathy: Macrophage modulation and tendon regeneration with injectable tendon hydrogel and tendon-derived stem cells exosomes (*Bioactive materials*)	2025	(41)	Cardiac repair using regenerating neonatal heart tissue-derived extracellular vesicles (*Nature Communications*)	2025	(42)
Self-Adaptive Release of Stem Cell-Derived Exosomes from a Multifunctional Hydrogel for Accelerating MRSA-Infected Diabetic Wound Repair (*Journal of the American Chemical Society*)	2025	(43)	Lemon-derived nanoparticle-functionalized hydrogels regulate macrophage reprogramming to promote diabetic wound healing (*Journal of Nanobiotechnology*)	2025	(44)
Self-healing adhesive oxidized guar gum hydrogel loaded with mesenchymal stem cell exosomes for corneal wound healing (*Journal of Nanobiotechnology*)	2025	(45)	Immunomodulation effects of collagen hydrogel encapsulating extracellular vesicles derived from calcium silicate stimulated-adipose mesenchymal stem cells for diabetic healing (*Journal of Nanobiotechnology*)	2025	(46)
A Bio-Responsive Hydrogel with Spatially Heterogeneous Structure for Treating Infectious Tissue Injuries (*Advanced Science*)	2025	(47)	The efficacy of platelet-derived extracellular vesicles in the treatment of diabetic wounds: a systematic review and meta-analysis of animal studies (*Archives of Dermatological Research*)	2025	(48)

**Figure 6:**
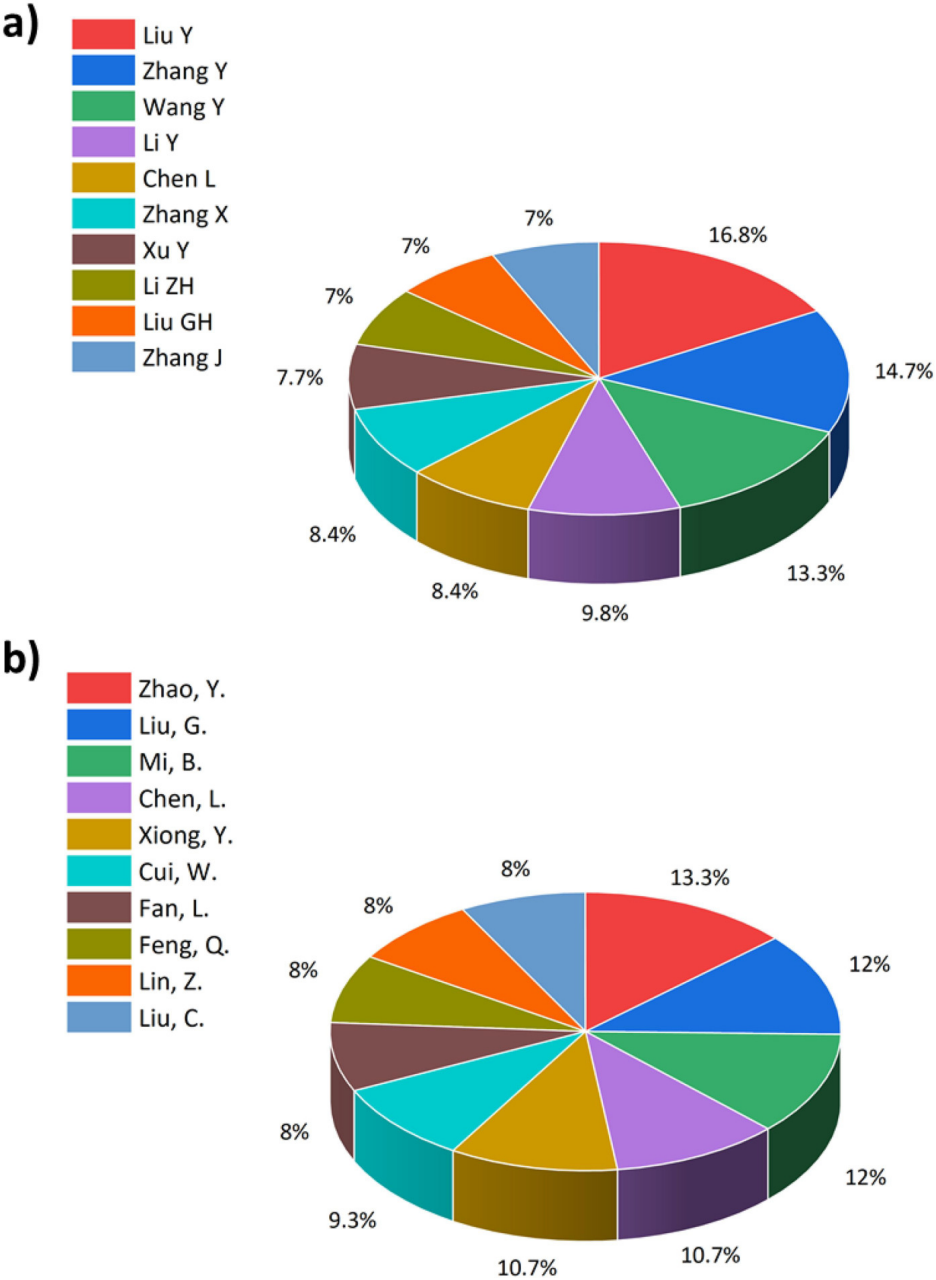
Top 10 authors listed with the highest record of publication based on: **(a)** WOS. **(b)** Scopus.

Figures 6a,b present the top 10 authors with respect to the record of the number of publications. According to the WOS database, Liu Y is the author with the highest contribution in this field, with a contribution accounting for 16.8% of the top 10-authors list. However, according to the Scopus database, Zhao Y. is the author with the highest record of publications, with a contribution of 13.3% to the list in the field of interest.

Although there are differences between the list exported from the WOS database and the Scopus database, it is important to point out that several authors are common between the two databases at higher rankings due to the difference in the number of publications recorded in each database.

d. Analysis of Keywords

**Figure 7:**
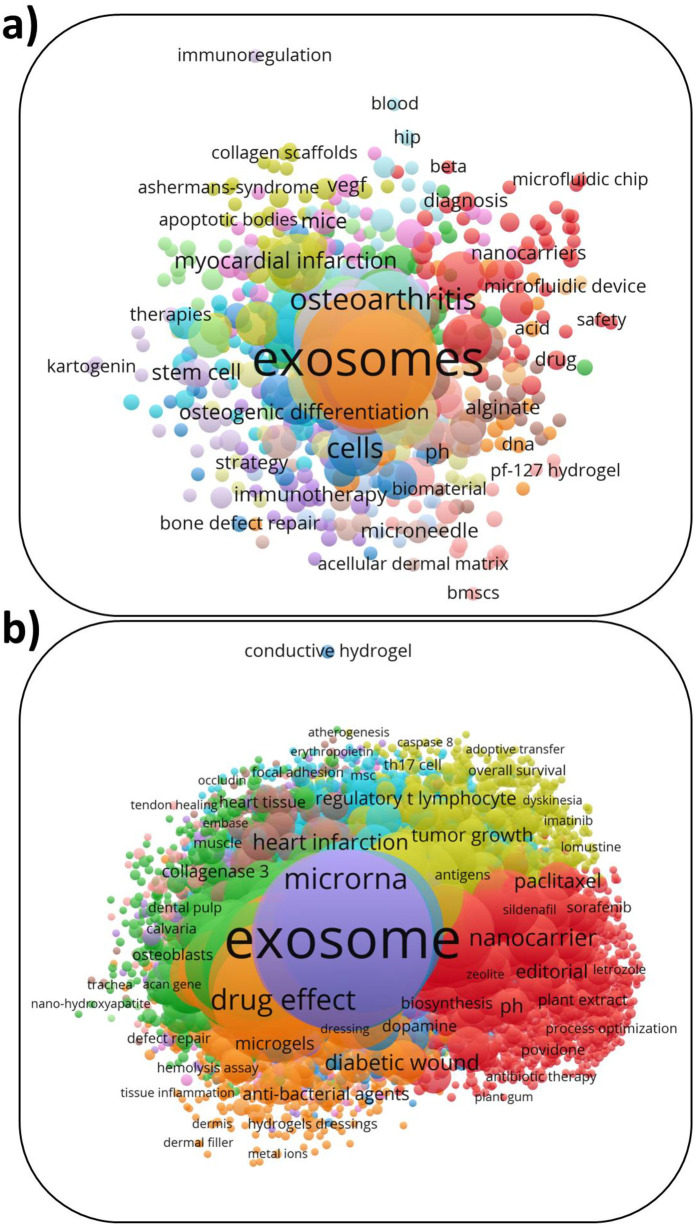
Co-occurrence keywords in the publications based on: **(a)** WOS. **(b)** Scopus.

The VOSviewer software was used to analyze the occurrence of keywords in the publications of both databases. The data were extracted from the databases and imported into the software as to perform the analyses. For the data exported from the WOS database, a threshold of 2 occurrences was used in addition to the identifying mechanism being “All Keywords”, which includes Author Keywords and Keywords Plus. Similarly, for the data exported from the Scopus database, a threshold of 2 occurrences was used in addition to the identifying mechanism being “All Keywords”, which includes Author Keywords and Indexed Keywords. For both datasets, the counting method was set to full counting. It is important to point out that due to the restriction of the WOS database in extraction, in the analysis of WOS keywords, only the first 500 publications were used. The maps presented in Figures 7a,b represent the overall interrelation of the keywords in the WOS and Scopus databases, respectively. The top 5 keywords with the highest frequency extracted from both databases are presented in Table 6. The words exosomes, hydrogel, and exosomes are of the highest frequency in both databases, which is consistent with the initial search criteria. The WOS database list includes Extracellular vesicles and Angiogenesis. While the Scopus database includes the words human, nonhuman, and humans, which are most probably used to describe the testing of the hydrogels, such as nonhuman specimens, for example, rats and mice.

**Table 6:** The top five keywords in the publications based on Scopus and WOS databases.

WOS			Scopus		
Keyword	Occurrences	Total link strength	Keyword	Occurrences	Total link strength
Exosomes	202	1,405	Exosome	1,135	58,272
Hydrogel	133	966	Hydrogel	1,120	57,544
Exosome	114	740	Human	914	49,189
Extracellular vesicles	92	746	Nonhuman	740	43,436
Angiogenesis	77	608	Humans	554	30,342

## 4 Conclusion

This study analysed the global research trends of the utilization of exosomes in hydrogels for various applications from 2015 to 9th–11th of May 2025 using data exported from the databases of WOS and Scopus. The data obtained reveal that a significant increasing trend exists in both the number of publications and citations in the field over the past decade, indicating a growth in the interest of this field in academic and clinical research. China has been the most dominant contributor to this field in terms of the number of publications, with several Chinese institutions affiliated with the most effective publications in the field. The publication with the highest citation count received 651 and 692 citations based on the WOS and Scopus databases. This record of citation is relatively average compared to other fields but notably high for this relatively new field of study. The main research areas that contribute to this field include material science, engineering, science and technology, and chemistry, which reflects the multidisciplinary nature of the exosome-hydrogel research field. This study provides an overview of the current evolution of research in exosome-hydrogel applications over the past decade and provides convenient insights for further development in this field in the future. Future work could expand the search to include additional types of EVs, such as macrovesicles and apoptotic bodies, and analyze them separately. Furthermore, carrier systems apart from hydrogels, such as nanoshells, can be explored.

## Data Availability

The original contributions presented in the study are included in the article/Supplementary Material, further inquiries can be directed to the corresponding authors.
